# Postexposure prophylaxis for HIV among healthcare workers in Türkiye: a descriptive, multicenter retrospective study

**DOI:** 10.55730/1300-0144.6186

**Published:** 2026-01-29

**Authors:** Veysel AKCA, İlkay AKBULUT, Gül Ruhsar YILMAZ, Alper TAHMAZ, Nefise ÖZTOPRAK ÇUVALCI, İlknur ESEN YILDIZ, Mehmet ÇABALAK, Şeyma TOPAL, Özgür GÜNAL, Bircan KAYAASLAN, Fatma ESER, Tuba DAMAR ÇAKIRCA, Deniz ÖZER, Aliye BAŞTUĞ, Firdevs AKSOY, Hanife Nur KARAKOÇ PARLAYAN, Beyza SÜLLÜ, Damla VERENDAĞ, Hatun ÖZTÜRK ÇERİK, Mehmet Ali AŞAN, Ayhan AKBULUT, Gülden ESER KARLIDAĞ, Behice KURTARAN, Hüsnü PULLUKÇU, Turhan TOGAN

**Affiliations:** 1 Department of Infectious Diseases and Clinical Microbiology, Sandıklı State Hospital, Afyonkarahisar, Turkiye; 2 Department of Infectious Diseases and Clinical Microbiology, Tepecik Training and Research Hospital, University of Health Sciences, İzmir, Turkiye; 3 Department of Infectious Diseases and Clinical Microbiology, Faculty of Medicine, Süleyman Demirel University, Isparta, Turkiye; 4 Department of Infectious Diseases and Clinical Microbiology, Antalya Training and Research Hospital, Antalya, Turkiye; 5 Department of Infectious Diseases and Clinical Microbiology, Faculty of Medicine, Recep Tayyip Erdoğan University, Rize, Turkiye; 6 Department of Infectious Diseases and Clinical Microbiology, Faculty of Medicine, Hatay Mustafa Kemal University, Hatay, Turkiye; 7 Department of Infectious Diseases and Clinical Microbiology, Kahramanmaraş State Hospital, Kahramanmaraş, Turkiye; 8 Department of Infectious Diseases and Clinical Microbiology, Faculty of Medicine, Samsun University, Samsun, Turkiye; 9 Department of Infectious Diseases and Clinical Microbiology, Faculty of Medicine, Ankara Yıldırım Beyazıt University, Ankara, Turkiye; 10 Department of Infectious Diseases and Clinical Microbiology, Faculty of Medicine, Harran University, Şanlıurfa, Turkiye; 11 Department of Infectious Diseases and Clinical Microbiology, Faculty of Medicine, Manisa Celal Bayar University, Manisa, Turkiye; 12 Department of Infectious Diseases and Clinical Microbiology, Ankara Bilkent City Hospital, University of Health Sciences Turkiye, Ankara, Turkiye; 13 Department of Infectious Diseases and Clinical Microbiology, Faculty of Medicine, Karadeniz Technical University, Trabzon, Turkiye; 14 Department of Infectious Diseases and Clinical Microbiology, Faculty of Medicine, Ege University, İzmir, Turkiye; 15 Department of Infectious Diseases and Clinical Microbiology, Faculty of Medicine, Ordu University, Ordu, Turkiye; 16 Department of Infectious Diseases and Clinical Microbiology, Adıyaman Training and Research Hospital, Adıyaman, Turkiye; 17 Department of Infectious Diseases and Clinical Microbiology, Faculty of Medicine, Fırat University, Elazığ, Turkiye; 18 Department of Infectious Diseases and Clinical Microbiology, Elazığ Fethi Sekin City Hospital, University of Health Sciences Turkiye, Elazığ, Turkiye; 19 Department of Infectious Diseases and Clinical Microbiology, Acıbadem Adana Hospital, Adana, Turkiye; 20 Department of Infectious Diseases and Clinical Microbiology, Faculty of Medicine, Muğla Sıtkı Koçman University, Muğla, Turkiye

**Keywords:** Healthcare workers, occupational exposure, needlestick injury, human immunodeficiency virus, postexposure prophylaxis, integrase inhibitor

## Abstract

**Background/aim:**

Postexposure prophylaxis (PEP) for human immunodeficiency virus (HIV) plays a vital role in preventing transmission among healthcare workers following occupational exposure. Despite its clinical importance, epidemiological data regarding PEP implementation in Türkiye remain scarce. This study aimed to assess PEP practices, prophylactic treatment regimens, and follow-up outcomes among healthcare workers at risk of occupational HIV exposure.

**Materials and methods:**

This retrospective, multicenter study was conducted between January 2020 and December 2024 across 16 healthcare facilities participating in the National HIV/AIDS Working Group. Healthcare workers aged 18 years and older who presented for evaluation following occupational exposure to HIV were included. Data on demographics, exposure characteristics, source patient test results, PEP initiation timing, regimen preferences, adverse effects, and follow-up outcomes were collected from hospital records and analyzed using SPSS v25.0.

**Results:**

A total of 369 healthcare workers were assessed. The PEP initiation rate was 49.1%. Needlestick injuries accounted for 79.4% of occupational exposures, and nurses constituted nearly half of presentations (49.6%). In univariable analyses, PEP initiation was less frequent among nurses than among other healthcare staff. In the multivariable model, male sex and age ≥40 years were independently associated with higher odds of PEP initiation, whereas the nursing profession was associated with lower odds. The median time to PEP initiation was 17.9 h (range: 0–120). Adverse effects were uncommon (3.5%), most frequently nausea and vomiting. No HIV seroconversions were identified among individuals with available follow-up results; however, follow-up testing at the predefined time points was incomplete, which limits the ability to exclude rare late seroconversions.

**Conclusion:**

Timely initiation of HIV PEP appears feasible and well tolerated in real-world occupational settings. These findings highlight the need for standardized institutional protocols and improved follow-up strategies for healthcare workers in Türkiye.

## 1. Introduction

Healthcare workers are at risk of occupational exposure to a wide range of infectious diseases. While established prophylactic measures are available for pathogens such as *Neisseria meningitidis*, hepatitis B virus, and rabies, human immunodeficiency virus (HIV) remains one of the most concerning due to its lifelong implications and transmission potential [1].^1^

According to the Turkish Ministry of Health, the number of HIV cases has shown a significant upward trend in recent years.^2^ Although institutional and anecdotal reports highlight the occurrence of occupational exposures, no national data have yet been published. The first studies on HIV postexposure prophylaxis (PEP) in the late 1990s demonstrated that zidovudine alone reduced the risk of occupational HIV transmission by approximately 80% [2].

High-risk exposures include percutaneous injuries, contact with mucous membranes, or contact with nonintact skin having infectious fluids such as blood, semen, vaginal secretions, or cerebrospinal fluid [3]. PEP decision-making is guided by the HIV status of the source patient and the type of exposure [4]. PEP is recommended in moderate- to high-risk exposures, ideally initiated within 72 h, and continued with a 28-day antiretroviral regimen [5]. However, in cases where presentation occurs beyond 72 h, PEP may still be considered in selected high-risk occupational exposures, based on specialist clinical judgment and an individualized risk assessment [6,7].

In occupational exposure settings, a negative HIV ribonucleic acid (RNA) test result in the source patient does not necessarily indicate zero transmission risk. During acute or early HIV infection, diagnostic test results may be negative due to the window period, and viral dynamics at the time of exposure may remain uncertain. Furthermore, the “undetectable = untransmittable” concept is based on evidence from sexual transmission under sustained viral suppression with antiretroviral therapy and cannot be directly extrapolated to percutaneous or mucocutaneous occupational exposures. Therefore, when acute HIV infection is clinically suspected in the source patient, initiation of PEP should be considered while diagnostic evaluation, including HIV RNA testing, is ongoing.^3^,^4^ After prophylaxis, HIV serological tests are conducted at predetermined times to monitor for potential seroconversion or to evaluate whether treatment should be stopped. Additionally, some individuals might need extra diagnostic tests. For instance, pregnancy screening is advised for female healthcare workers.^5^ It is important to regularly assess aspartate aminotransferase (AST), alanine aminotransferase (ALT), and creatinine levels to detect potential medication-related adverse effects [8,9].

Despite the increasing number of cases, epidemiological data on occupational HIV exposure and PEP utilization in Türkiye remain scarce. To address this gap, this study was designed as a multicenter retrospective study across Türkiye to contribute to the existing scientific literature.

## 2. Materials and methods

### 2.1. Study design

This multicenter retrospective descriptive study was carried out from January 1, 2020, to December 30, 2024, by reviewing medical records. The study included individuals evaluated for HIV PEP after occupational exposure at the Infectious Diseases and Clinical Microbiology clinics of the participating centers. A standardized data collection form was used to extract information from each center’s electronic medical record system and/or printed archives.

### 2.2. Inclusion criteria

- Healthcare workers aged 18 years and above
- Individuals who applied for evaluation following occupational exposure to HIV for consideration of PEP
- Individuals evaluated according to the standardized institutional postexposure assessment protocol for occupational HIV exposure at the participating centers during the study period

### 2.3. Variables

Demographic characteristics (age, sex, profession), exposure-related variables (type of exposure, HIV serostatus of the source, and HIV RNA level when available), time to presentation, time to initiation of postexposure prophylaxis, initiation of HIV PEP, antiretroviral regimen used for prophylaxis, occurrence of adverse effects, follow-up laboratory and serological test results, and baseline and follow-up biochemical parameters (ALT, AST, creatinine) were recorded as analytical variables. Age was analyzed as both a continuous and a categorical variable. Continuous age was used for descriptive analyses, while age was categorized into clinically relevant groups (18–30, 30–40, and ≥40 years) for comparative analyses and multivariable logistic regression to improve interpretability and avoid assuming strict linearity. Sensitivity analyses using continuous age and alternative categorizations yielded consistent results, and the three-category grouping was retained. Nurses were analyzed separately because they constituted a large, operationally distinct group, allowing for more interpretable multivariable comparisons.

### 2.4. Definitions

Occupational exposure was defined as any percutaneous, mucocutaneous, or nonintact skin contact that required evaluation for HIV PEP based on the nature of the exposure and potential risk of transmission. This evaluation was performed even when the source patient’s anti-HIV test result was negative, considering that the HIV serostatus at the time of exposure may be unknown, outdated, or require subsequent confirmation. A confirmed case was defined as an individual with HIV positivity verified by confirmatory testing following a reactive screening test, in accordance with international guidelines [2,6,7]. Reported postexposure test results were limited to follow-up evaluations performed at weeks 2, 4, 6, and 12 after exposure.

### 2.5. Statistical analysis

Descriptive statistics were used to summarize the data. Categorical variables were expressed as frequencies and percentages. Continuous variables were reported as means with standard deviations or as medians with ranges, depending on their distribution.

Comparisons between groups were performed using the Pearson chi-square test for categorical variables. Time to initiation of PEP was treated as a nonnormally distributed variable and summarized using the median and range.

Binary logistic regression analysis was performed to identify factors independently associated with initiation of HIV PEP. The dependent variable was PEP initiation (yes/no). Variables entered into the multivariable logistic regression model were selected based on clinical relevance and results from the univariate analyses. Source patient HIV serostatus, although strongly associated with PEP initiation, was not included in the multivariable model because it constitutes a proximal determinant of the clinical decision to initiate PEP and its inclusion could lead to overadjustment. Odds ratios (ORs) with 95% confidence intervals (95% CI) were calculated. Model fit was assessed using the Hosmer–Lemeshow goodness-of-fit test.

Missing data were handled using complete-case analysis. Participants with missing data for variables included in a given analysis were excluded from that specific analysis. No imputation methods were applied. Baseline demographic characteristics appeared broadly similar between participants with complete follow-up data and those with missing follow-up data. Sensitivity analyses were conducted by modeling age as a continuous variable and using alternative age categorizations to assess the robustness of the findings.

A two-sided p-value of <0.05 was considered statistically significant. All statistical analyses were performed using IBM SPSS Statistics, v25.0 (IBM Corp., Armonk, NY, USA).

### 2.6. Ethical approval

The study protocol was approved by the institutional ethics committee (Protocol No: 250028, Decision No: 65, March 14, 2025). The study was conducted in accordance with the principles of the Declaration of Helsinki. As this was a retrospective review of anonymized medical records, the requirement for obtaining informed consent was waived by the ethics committee. No external funding was received.

## 3. Results

A total of 369 healthcare workers from 16 centers were evaluated for HIV PEP. While all eligible participants were included in the study, treatment-related analyses were conducted on 367 participants due to refusal of PEP by two individuals. Baseline demographic characteristics and exposure types are summarized in Table 1. Source patient HIV status, available HIV RNA levels, and accompanying hepatitis and syphilis serologies are presented in Table 2. Baseline serological screening results, hepatitis B immunity status, and pregnancy testing are summarized in Table 3.

The median time to initiation was 17.9 h (range: 0–120). PEP was initiated in 181 healthcare workers (49.1%), while two individuals (0.5%) refused prophylaxis. Adverse effects were reported in 3.5% of participants, most commonly nausea and vomiting. One individual (0.5%) required a regimen change due to nausea and vomiting. Prophylaxis was switched from bictegravir/emtricitabine/tenofovir alafenamide (BIC/FTC/TAF) to dolutegravir/emtricitabine/tenofovir disoproxil fumarate (DTG/FTC/TDF). Regimens were predominantly integrase inhibitor-based, and adverse events were uncommon and generally mild (Table 4).

Anti-HIV serology and, when available, HIV RNA testing were scheduled at weeks 4, 6, and 12; however, follow-up testing at these time points was incomplete (Table 5). Among participants with available follow-up results, no HIV seroconversions were identified. Additional anti-HIV tests recorded beyond the predefined visits were available for some individuals and were also negative; nevertheless, incomplete follow-up remains a key limitation for excluding rare late seroconversions.

Group comparisons for PEP initiation by demographic and exposure characteristics, including source patient HIV status, are presented in Table 6. The distribution of time to PEP initiation across demographic and exposure characteristics is shown in Table 7. In multivariable logistic regression, male sex and age ≥40 years were independently associated with higher odds of PEP initiation, whereas nursing profession was associated with lower odds. Fluid exposure to nonintact skin was associated with increased odds of PEP initiation compared with needlestick injury (Table 8). Source patient HIV status also differed significantly by profession, with nurses having lower rates of HIV-positive or viremic source exposures (p < 0.001).

## 4. Discussion

### 4.1. Types of exposure

A systematic review and metaanalysis published in the WHO Eastern Mediterranean Health Journal in 2022 reported that approximately 43% of healthcare workers worldwide had experienced at least one needlestick injury during their careers (95% CI: 37%–49%). The prevalence was higher in Africa, reaching 51% (95% CI: 40%–61%), with needle recapping and hollow-bore needles identified as primary causes [10]. Other metaanalyses reported lifetime prevalence rates of up to 56.2% (95% CI: 47.1%–64.9%) and 32.4% in the previous year (95% CI: 22.0%–44.8%) [11]. Furthermore, exposures due to blood splashes remain a significant contributor, as highlighted in studies from Ethiopia where 42.9% of workers experienced needlestick injuries and 31% reported blood splashes [12].

In this study, needlestick injuries accounted for 79.4% of all exposures, a proportion considerably higher than the global estimates. This discrepancy may reflect the hospital-based and PEP-indicated nature of our cohort. Although exposure sites were not recorded in detail, previous studies have shown that intensive care units and operating rooms are among the most common high-risk settings [13,14]. Notably, the findings also emphasize that nonpercutaneous exposures such as blood splashes, though less frequent, represent nonnegligible risks.

### 4.2. PEP initiation rates and timing

A PEP initiation rate of 49.1% was observed in this study, lower than the rates reported in several international studies. For example, a study from Zimbabwe reported initiation rates exceeding 80%, though treatment completion remained poor [4]. In Ghana, only 65% of healthcare workers who started PEP completed the regimen [5]. In contrast, earlier Italian multicenter data highlight the occupational risk context in healthcare settings [15].

The relatively low PEP initiation rate observed in Türkiye may reflect several factors, including limited awareness of occupational exposure risks and the relatively low perceived prevalence of infection when the source patient’s serostatus is unknown. Nevertheless, adherence among individuals who initiated PEP was high; early discontinuation observed in three participants occurred only after confirmation that the source patient was HIV negative and therefore reflected an appropriate risk-based clinical decision rather than poor adherence [7].

International guidelines recommend that PEP should ideally be initiated as soon as possible after exposure, preferably within the first 2 h and no later than 72 h. In this study’s cohort, the median time to PEP initiation was 17.9 h (range: 0–120). When categorized according to guideline-relevant thresholds, most participants initiated prophylaxis within 24 h, while a smaller proportion experienced delays beyond 24 h. Although the overall initiation times fall within the recommended window, these findings highlight clinically relevant delays when compared with the optimal timeframe. Previous studies have associated such delays with limited awareness, inefficiencies in referral pathways, and restricted access to medications outside standard working hours [16–18]. These findings suggest that system-level factors, including variability in institutional protocols and access to postexposure services, may contribute to delayed or reduced PEP initiation in Türkiye.

Comparisons between healthcare workers who did and did not initiate HIV PEP indicate that the decision to initiate PEP was influenced by multiple demographic and exposure-related factors. PEP initiation was more frequent among male healthcare workers and among those aged ≥40 years. In contrast, nurses demonstrated significantly lower initiation rates compared with other healthcare staff. Similar profession-related differences in PEP utilization have been reported in previous studies and may be related to differences in occupational roles, workload intensity, and access to timely specialist evaluation [4,12]. As expected, PEP initiation was strongly associated with the HIV serostatus of the source patient, with substantially higher uptake when the source was HIV-positive or had an uncertain serostatus, underscoring the central role of source-related risk assessment in clinical decision-making [6,7].

Subgroup analyses provided additional insight into factors associated with delays in HIV PEP initiation. Delays beyond 24 h were more frequently observed among older healthcare workers. This finding is consistent with previous reports from different healthcare settings and may be related to differences in risk perception among more experienced staff, underestimation of exposure severity, or delayed presentation for evaluation [4,5].

Profession-related differences in PEP initiation timing were also evident. Nurses demonstrated a distinct timing pattern compared with other healthcare workers. This may be influenced by high workload, shift-based working conditions, and potential delays in accessing occupational health or infectious diseases services, particularly outside regular working hours. Similar profession-related delays and variations in PEP utilization have been described in comparable healthcare settings [4,12].

Timing of PEP initiation also varied according to the type of exposure. A higher proportion of delayed initiation was observed following mucous membrane exposures compared with needlestick injuries. This finding may reflect the perception of nonpercutaneous exposures as lower risk events, which can reduce the perceived urgency to initiate PEP. Previous studies have similarly reported delayed or reduced PEP use following mucosal or splash-type exposures [3,16,17].

No statistically significant difference in PEP initiation timing was observed between sexes, suggesting that delays in PEP initiation are more strongly influenced by occupational role, exposure characteristics, and structural factors related to healthcare access rather than by sex-specific determinants.

The multivariable logistic regression analysis (Table 8) identified factors independently associated with initiation of HIV PEP. Male sex and older age (≥40 years) were independently associated with higher odds of PEP initiation after adjustment for relevant covariates. Similar associations between demographic characteristics and PEP uptake have been reported previously, suggesting that perceived exposure severity, risk appraisal, and clinical experience may influence decisions regarding prophylaxis initiation [4,5]. The nursing profession was independently associated with lower odds of PEP initiation. This association may partly reflect a higher proportion of exposures involving HIV-negative source patients among nurses and a consequently lower perceived transmission risk, as well as operational factors such as workload, shift-based working patterns, and barriers to timely access to specialist consultation. Consistent with findings from comparable healthcare settings, nurses may therefore be less likely to initiate or complete PEP, underscoring the need for targeted institutional interventions to improve risk assessment, timely reporting, and adherence to recommended prophylaxis protocols [4,12].

With respect to exposure characteristics, fluid exposure to nonintact skin was independently associated with higher odds of PEP initiation compared with needlestick injuries. This observation aligns with prior studies suggesting that exposures perceived as involving larger volumes of potentially infectious material may prompt a stronger clinical response [3,6]. In contrast, mucous membrane exposures were not independently associated with PEP initiation after adjustment, suggesting that such exposures may be variably perceived in terms of transmission risk in real-world practice.

### 4.3. Preferred regimens and side effects

The most frequently prescribed regimen in this study was DTG/FTC/TDF, consistent with current guidelines and global practice [1,6,7]. Across Europe, integrase strand transfer inhibitor (INSTI)-based combinations, especially dolutegravir- or bictegravir-containing regimens, are increasingly favored [1]. In addition to these two regimens, other INSTIs such as raltegravir (RAL) and elvitegravir (EVG) have also been used for HIV PEP. RAL-based PEP has demonstrated favorable tolerability, fewer drug–drug interactions, and improved adherence in real-life clinical settings. Similarly, single-tablet EVG/cobicistat (COBI)/FTC/TAF regimens have shown acceptable safety and efficacy profiles in clinical studies of HIV PEP. The use of these regimens in our cohort likely reflects real-world practice, including temporal changes in guideline recommendations and variability in drug availability across centers [19,20].

Adverse effects were infrequent in this study, observed in only 3.5% of participants, primarily nausea and vomiting. Gan et al. reported adverse event rates of 15.2% for BIC/FTC/TAF and 10.3% for TDF/FTC + DTG, with treatment discontinuation in only 1.1% of cases [21].

In contrast, no participants in this study discontinued therapy due to side effects, supporting the observed tolerability of INSTI-based regimens in this cohort. Adherence among individuals who initiated PEP was high; three participants discontinued prophylaxis early after confirmation that the source patient was HIV negative, reflecting appropriate risk-based clinical decision-making rather than intolerance. Despite this overall favorable safety and tolerability profile, variability in PEP regimen selection was observed across centers. This heterogeneity likely reflects real-world practice rather than inconsistent clinical decision-making, and may be influenced by differences in institutional protocols, updates in national guideline recommendations over time, variability in antiretroviral drug availability within hospital formularies, reimbursement policies, and clinician familiarity with specific regimens. Such system-level factors are inherent to multicenter, real-world studies and should be considered when interpreting regimen heterogeneity. In Türkiye, PEP regimen choice is influenced by institutional formulary availability and reimbursement mechanisms. National guidance lists a tenofovir/emtricitabine backbone (TDF/FTC or TAF/FTC) combined with an integrase inhibitor (RAL or DTG) as preferred options, with alternative integrase- or boosted-PI–based combinations, such as EVG/COBI/TDF/FTC, EVG/COBI/TAF/FTC, DTG + TDF/FTC, DTG + TAF/FTC, BIC/TAF/FTC, or DRV/r-based regimens, depending on availability and clinical context. In this study’s multicenter cohort, the most commonly used regimens followed this backbone + INSTI approach, while alternative combinations were used in a minority of cases [22].

### 4.4. Follow-up and serological testing

A critical issue identified in this study was the lack of consistent serological monitoring. A substantial proportion of participants had missing results at the 4th, 6th, and 12th weeks, echoing global challenges. For example, in Zimbabwe, follow-up rates after PEP initiation were only 6% at week 6, 2% at month 3, and less than 1% at month 6 [4]. The Southern African HIV Clinicians Society’s 2023 guideline similarly highlighted high initiation but low completion and monitoring rates [23].

While no seroconversions were observed in this study’s cohort, incomplete follow-up raises concerns about the potential to miss late seroconversions. This pattern has also been reported in other settings [4]. Factors likely contributing to inadequate follow-up include limited institutional infrastructure, absence of reminder systems, heavy workloads, and insufficient awareness [24,25].

Although corrective strategies were not investigated for this study, prior studies suggest that digital reminders, automated scheduling, and stronger institutional oversight may improve adherence to monitoring protocols [26].^6^ These findings suggest that strengthening national guidelines and institutional systems to address not only initiation but also consistent completion and follow-up appears warranted [23,25].

Postexposure evaluation should not be limited to HIV alone but should also include assessment for other blood-borne and sexually transmitted pathogens, particularly in the context of potential coinfections. Current guidelines recommend evaluation for hepatitis B, hepatitis C, and syphilis as part of a comprehensive postexposure approach. In this context, anti-HCV, HBsAg, anti-HBc IgG, and syphilis serological test results were also evaluated in this study to reflect a broader, real-world postexposure assessment framework [27,28].

Recent national studies from Türkiye have also highlighted challenges in translating guideline recommendations into consistent clinical practice, supporting this study’s findings regarding incomplete follow-up and monitoring [29–31].

## 5. Conclusion

This study represents the first multicenter investigation in Türkiye evaluating occupational HIV exposures and the use of PEP among healthcare workers. Although no HIV seroconversions were identified based on available follow-up testing, including anti-HIV evaluations performed beyond the predefined follow-up visits, delays in initial evaluation, limited PEP initiation rates, and gaps in serological monitoring were observed. Most PEP initiations occurred within the first 24 h following exposure, indicating a generally timely clinical response in this cohort. However, the observed gaps in initiation and follow-up underline the importance of improving timely access to care, strengthening standardized institutional protocols, and ensuring consistent follow-up practices. Overall, this study provides multicenter data from Türkiye and highlights key areas of occupational HIV exposure management that may benefit from improvement at the system level.

## 6. Study limitations

This study’s retrospective design and reliance on existing medical record systems resulted in missing data for certain parameters. In particular, information on biochemical markers such as ALT, AST, and creatinine, as well as pregnancy screening outcomes among female healthcare workers, was often incomplete.

Although serological testing at predefined follow-up time points was inconsistent, available anti-HIV test results obtained at later time points did not demonstrate HIV seroconversion in this cohort. However, incomplete and nonstandardized follow-up represents a major limitation and may have reduced the exclusion of rare late seroconversions.

In addition, the multicenter nature of the study may have introduced variability in data quality across participating centers. These limitations should be considered when interpreting the findings, and future studies should be designed prospectively using standardized protocols to ensure more complete follow-up and data consistency.

## 7. Strengths of the study

This study provides a large multicenter dataset on HIV PEP practices among healthcare workers in Türkiye (16 centers, 369 cases). Its real-world design offers insights into presentation delays, gaps in follow-up testing, and regimen tolerability. These findings may inform institutional protocols and public health strategies aimed at improving the management of occupational exposures.

## Figures and Tables

**Table 1:** Demographic and exposure characteristics of healthcare workers evaluated for HIV PEP.

	n	%	Mean ± SD

Age (years) (Mean ± SD)			32.7 ± 9.4

Age groups (years)			
18–30 years	214	58.0	
30–40 years	70	19.0	
40+ years	85	23.0	

Sex			
Female	262	71.0	
Male	107	29.0	

Profession			
Physician	106	28.7	
Nurse	183	49.6	
Paramedic (emergency technician)	9	2.4	
Allied healthcare workers	36	9.8	
Cleaning staff	32	8.7	
Other	3	0.8	

Type of exposure			
Needlestick injuries	293	79.4	
Fluid exposure to nonintact skin	22	6.0	
Fluid exposure to mucous membranes	54	14.6	

Values are presented as mean ± standard deviation (SD) or number (%). PEP = postexposure prophylaxis. The “Other” category includes healthcare workers who could not be classified as physicians, nurses, paramedics, allied healthcare workers, or cleaning staff (e.g., administrative or technical personnel).

**Table 2:** Serological characteristics of source patients associated with occupational HIV exposure.

	n	%	Median (range)

HIV serostatus			
Negative	148	40.1	
Positive (with or without detectable viremia)	118	32.0	
Awaiting confirmatory testing	33	8.9	
Unknown	70	19.0	

HIV RNA level (IU/mL) (n = 62, median, range)			11,700 (0–4,000,000)

Anti-HCV status (n = 342)			
Negative	231	67.5	
Positive	30	8.8	
Not tested	81	23.7	

HBsAg status (n = 342)			
Negative	237	69.3	
Positive	21	6.1	
Not tested	84	24.6	

Anti-HBc IgG status (n = 341)			
Negative	139	40.8	
Positive	33	9.6	
Not tested	169	49.6	

Anti-HBs status (n = 342)			
Negative	139	40.6	
Positive	93	27.2	
Not tested	110	32.2	

Syphilis serology (n = 340)			
Negative	57	16.8	
Positive	13	3.8	
Not tested	270	79.4	

Values are presented as number (percentage) unless otherwise specified. HIV RNA levels are expressed as median (range) and are presented as IU/mL.

**Table 3:** Serological and pregnancy characteristics of healthcare workers assessed for HIV PEP.

	n	%

HIV status (n = 369)		
Negative	363	98.4
Not tested	6	1.6

Anti-HCV result (n = 367)		
Negative	364	99.2
Positive	2	0.5
Not tested	1	0.3

HBsAg result (n = 368)		
Negative	328	89.1
Positive	32	8.7
Not tested	8	2.2

Anti-HBc IgG result (n = 341)		
Negative	255	69.7
Positive	14	3.8
Not tested	97	26.5

Anti-HBs result (n = 342)		
Negative	26	7.1
Positive	325	88.5
Not tested	16	4.4

Syphilis result (n = 341)		
Negative	102	28.1
Positive	3	0.8
Not tested	258	71.1

Pregnancy status (n = 262)		
Negative	76	29.0
Positive	1	0.4
Not tested	185	70.6

Values are expressed as number (percentage).

**Table 4:** PEP characteristics of healthcare workers.

	n	%	median, range/mean ± SD

Delay in initial evaluation (h) (n = 349)			3 (0–120)

Decision to initiate prophylaxis (n = 369)			
No	184	49.9	
Yes	181	49.1	

Refused by individual	2	0.5	

Delay in initiating prophylaxis (h) (n = 163)			17.9 (0–120)

ART regimen initiated for prophylaxis			
1. DTG/FTC/TDF	118	65.2	
2. BIC/FTC/TAF	26	14.4	
3. EVG/COBI/FTC/TAF	6	3.4	
4. RAL/FTC/TDF	30	16.6	
5. RAL/FTC/TAF	1	0.4	

Presence of adverse effects after prophylaxis (n = 178)			
No	170	95.5	
Nausea and vomiting	5	2.8	
Headache	3	1.7	

ART regimen changed due to side effects? (n = 177)			
No	176	99.5	
Yes	1	0.5	

Duration of prophylaxis (n = 178)			27.8 ± 4.91

Symptoms or findings of ARS during follow-up (n = 369)			
None	181	49.1	
Not assessed	188	50.9	

Reexposure while on PEP (n = 369)			
No	304	82.4	
Missing data	65	17.6	

History of previous PEP use (n = 369)			
No	309	83.7	
Yes	2	0.6	
Missing data	58	15.7	

Early discontinuation of prophylaxis	3		

Values are expressed as number (percentage), mean ± standard deviation (SD), or range as appropriate. ART = antiretroviral therapy, PEP = postexposure prophylaxis, DTG = dolutegravir, FTC = emtricitabine, TDF = tenofovir disoproxil fumarate, BIC = bictegravir, TAF = tenofovir alafenamide, RAL = raltegravir, EVG = elvitegravir, and COBI = cobicistat.

**Table 5:** Follow-up laboratory and serological results of healthcare workers assessed for HIV PEP.

	n	%	median, range / mean ± SD

Week 4 anti-HIV test result (n = 369)			
Negative	286	77.5	
Missing data	83	22.5	

Week 6 anti-HIV test result (n = 369)			
Negative	94	25.5	
Missing data	275	74.5	

Week 12 anti-HIV test result (n = 369)			
Negative	268	72.6	
Missing data	101	27.4	

Week 4 HIV RNA test result (n = 369)			
Negative	54	14.6	
Missing data	315	85.4	

Week 6 HIV RNA test result (n = 369)			
Negative	13	3.5	
Missing data	356	96.5	

Week 12 HIV RNA test result (n = 369)			
Negative	62	16.8	
Missing data	307	83.2	

Baseline creatinine (mg/dL) (n = 195)			0.75 ± 0.14

Baseline ALT (U/L) (n = 201)			19.97 ± 8.79

Baseline AST (U/L) (n = 201)			20.42 ±13.59

Week 2 ALT (U/L) (n = 18)			16 (9–48)

Week 2 AST (U/L) (n = 16)			17.5 (9.5–57)

Week 2 creatinine (mg/dL) (n = 19)			0.8 (0.57–1.3)

Week 4 ALT (U/L) (n = 58)			26.85 ± 15.04

Week 4 AST (U/L) (n = 59)			20.58 ± 7.80

Week 4 creatinine (mg/dL) (n = 59)			0.87 ± 0.17

Values are expressed as mean ± standard deviation (SD), median, range or number (percentage), as appropriate. ALT and AST levels are presented in U/L, and creatinine levels are presented in mg/dL.

**Table 6:** Factors associated with initiation of HIV PEP (n = 367).

	PEP not initiated	PEP initiated	p

Age groups (years)			
18–30 years (n = 214)	122^a^ (57.0)	94^b^ (43.0)	0.016*
30–40 years (n = 70)	28^a^ (40.6)	41^a^ (59.4)	
40+ years (n = 85)	36^a^ (42.9)	48^a^ (57.1)	

Sex			
Female (n = 261)	148^a^ (56.7)	113^b^ (43.3)	<0.001*
Male (n = 106)	38^a^ (35.8)	68^b^ (64.2)	

Profession			
Other healthcare staff (n = 184)	70^a^ (38.0)	114^b^ (62.0)	<0.001*
Nurse (n = 183)	116^a^ (63.4)	67^b^ (36.6)	

Type of exposure			
Needlestick injuries	158^a^ (54.3)	133^b^ (45.7)	0.014*
Fluid exposure to nonintact skin	6^a^ (27.3)	16^b^ (72.7)	
Fluid exposure to mucous membranes	22^a^ (40.7)	32^a^ (59.3)	

HIV serostatus			
Negative	146^a^ (98.6)	2^b^ (1.4)	<0.001*
Positive (with or without detectable viremia)	9^a^ (7.6)	109^b^ (92.4)	
Awaiting confirmatory testing	8^a^ (24.2)	25^b^ (75.8)	
Unknown	23^a^ (33.8)	45^b^ (66.2)	

Data are presented as number (%). Overall group comparisons were performed using the Pearson chi-square test. When the overall test was statistically significant, post hoc pairwise comparisons of column proportions were conducted with Bonferroni adjustment; different superscript letters within the same row indicate statistically significant pairwise differences. A p-value of <0.05 was considered statistically significant. Two healthcare workers who declined PEP were excluded from treatment-related analyses; therefore, the total number for this table is 367.

**Table 7:** Distribution of time to PEP initiation according to demographic and exposure characteristics.

	≤24 h	24–72 h	>72 h	p

Age groups (years)				
18–30 years (n = 214)	61^a^ (76.3)	17^b^ (21.3)	2^c^ (2.4)	
30–40 years (n = 70)	29^a^ (74.4)	3^a^ (7.7)	7^b^ (17.9)	0.009*
40+ years (n = 85)	37^a^ (84.1)	4^a^ (9.1)	3^a^ (6.8)	

Sex				
Female (n = 261)	78^a,b^ (78.0)	18^b^ (18.0)	4^a^ (4.0)	0.053*
Male (n = 106)	49^a,b^ (77.8)	6^b^ (9.5)	8^a^ (12.7)	

Profession				
Other healthcare staff (n = 184)	75^a^ (75.0)	13^a^ (13.0)	12^b^ (12.0)	0.015*
Nurse (n = 183)	52^a^ (82.5)	11^a^ (17.5)	0^b^ (0.0)	

Type of Exposure				
Needlestick injuries	102^a^ (85.7)	13^b^ (10.9)	4^b^ (3.4)	
Fluid exposure to nonintact skin	15^a^ (88.2)	2^a^ (11.8)	0^a^ (0.0)	<0.001*
Fluid exposure to mucous membranes	10^a^ (37.0)	9^b^ (33.3)	8^b^ (29.7)	

Data are presented as number (%). Time to initiation of PEP was categorized as ≤24 h, 24–72 h, and >72 h in accordance with guideline-relevant thresholds. Overall group comparisons were performed using the Pearson chi-square test. When the overall test was statistically significant, post hoc pairwise comparisons of column proportions were conducted with Bonferroni adjustment; different superscript letters within the same row indicate statistically significant pairwise differences. A p-value of <0.05 was considered statistically significant. Two healthcare workers who declined PEP were excluded from treatment-related analyses; therefore, the total number for this table is 367.

**Table 8:** Multivariable logistic regression analysis of factors associated with initiation of HIV PEP.

Variable	Odds ratio	95% CI	p-value

Sex			
Male vs female	1.77	1.05–2.99	0.031

Age group (ref: 18–30 years)			
30–40 years vs 18–30 years	1.54	0.82–2.88	0.152
≥40 years vs 18–30 years	1.95	1.13–3.36	0.016

Profession			
Nurse vs other healthcare staff	0.43	0.27–0.67	<0.001

Type of exposure (ref: needlestick injury)			
Fluid exposure to nonintact skin vs needlestick	4.10	1.49–11.28	0.006
Fluid exposure to mucous membranes vs needlestick	1.73	0.92–3.24	0.086

Binary logistic regression analysis was performed to identify healthcare worker- and exposure-related factors independently associated with initiation of HIV PEP. The dependent variable was PEP initiation (yes/no). Categorical independent variables (sex, age group, profession, and type of exposure) were entered into the model using dummy coding with predefined reference categories. The reference categories were female sex, age 18–30 years, other healthcare staff, and needlestick injury. Odds ratios (OR) with 95% confidence intervals (95% CI) are reported. A two-sided p-value of <0.05 was considered statistically significant.

## Data Availability

The datasets generated and/or analyzed during the current study are available from the corresponding author upon reasonable request, subject to institutional and confidentiality restrictions.

## Footnotes

1 Centers for Disease Control and Prevention (2024). Risk factors: infectious agents in healthcare settings [online]. https://www.cdc.gov/niosh/healthcare/risk-factors/infectious-agents.html [Accessed 27 September 2025].

2 Republic of Türkiye Ministry of Health, General Directorate of Public Health. HIV/AIDS Statistics. https://hsgm.saglik.gov.tr/depo/birimler/bulasici-hastaliklar-ve-erken-uyari-db/Dokumanlar/Istatistikler/HIV_AIDS_Istatistikleri.pdf [Accessed January 2026].

3 Centers for Disease Control and Prevention (CDC). HIV testing overview: window period. https://www.cdc.gov/hiv/testing/index.html [Accessed January 6, 2026].

4 National Clinician Consultation Center (NCCC). PEP quick guide for bloodborne pathogen exposures. Updated October 2025. https://nccc.ucsf.edu/clinical-resources/pep-resources/pep-quick-guide-for-exposures/ [Accessed January 6, 2026].

5 Centers for Disease Control and Prevention (CDC) (2023). Special populations: pregnant healthcare personnel [online]. https://www.cdc.gov/infection-control/hcp/healthcare-personnel-epidemiology-control/pregnant-hcp.html [Accessed 27 September 2025].

6 Centers for Disease Control and Prevention (CDC) (2025). Clinical guidance for HIV post-exposure prophylaxis [online]. https://www.cdc.gov/hivnexus/hcp/index.html [Accessed 27 September 2025].

## References

[1] Han A Henderson DK Postexposure prophylaxis for occupational exposure to selected pathogens for healthcare personnel Current Opinion in Infectious Diseases 2024 37 4 296 303 10.1097/QCO.0000000000001029 38899948 PMC11213494

[2] Beltrami EM Williams IT Shapiro CN Chamberland ME Risk and management of blood-borne infections in health care workers Clinical Microbiology Reviews 2000 13 3 385 407 10.1128/CMR.13.3.385-407.2000 10885983 PMC88939

[3] Pineda-Ramirez JL Sierra-Diaz E Zavala-Sánchez EV Zarate-Leal G Cisneros-García DL The prevalence of HIV seroconversion in healthcare workers following sharp injuries and exposure to biofluids Cureus 2024 16 8 e66773 10.7759/cureus.66773 39268289 PMC11392009

[4] Mushambi F Timire C Harries AD Tweya H Goverwa-Sibanda TP High post-exposure prophylaxis uptake but low completion rates and HIV testing follow-up in health workers, Harare, Zimbabwe Journal of Infection in Developing Countries 2021 15 4 559 565 10.3855/jidc.12214 33956657 PMC8655953

[5] Suglo RE Aku FY Anaman-Torgbor JA Tarkang EE Predictors of adherence to HIV post-exposure prophylaxis protocol among frontline healthcare workers at the Ho Teaching Hospital, Ghana International Journal of Infectious Diseases 2021 106 208 212 10.1016/j.ijid.2021.03.079 33812009

[6] Kuhar DT Henderson DK Struble KA Heneine W Thomas V Updated US Public Health Service guidelines for the management of occupational exposures to human immunodeficiency virus and recommendations for postexposure prophylaxis Infection Control and Hospital Epidemiology 2013 34 9 875 892 10.1086/672271 23917901

[7] Kofman AD Struble KA Heneine W Gayle B de Perio MA 2025 US Public Health Service guidelines for the management of occupational exposures to human immunodeficiency virus and recommendations for post-exposure prophylaxis in healthcare settings Infection Control and Hospital Epidemiology 2025 46 9 863 873 10.1017/ice.2025.10254 41569270 PMC12616222

[8] Otto AO Rivera CG Zeuli JD Temesgen Z Hepatotoxicity of contemporary antiretroviral drugs: a review and evaluation of published clinical data Cells 2021 10 5 1263 10.3390/cells10051263 34065305 PMC8160846

[9] Asirvatham ES Ranjan V Garg C Sarman CJ Periasamy M A review of tenofovir disoproxil fumarate associated nephrotoxicity among people living with HIV: burden, risk factors and solutions Clinical Epidemiology and Global Health 2024 25 101462 10.1016/j.cegh.2023.101462

[10] Hosseinipalangi Z Golmohammadi Z Ghashghaee A Ahmadi N Hosseinifard H Global, regional and national incidence and causes of needlestick injuries: a systematic review and meta-analysis Eastern Mediterranean Health Journal 2022 28 3 233 241 10.26719/emhj.22.031 35394056

[11] Mengistu DA Tolera ST Demmu YM Worldwide prevalence of occupational exposure to needle stick injury among healthcare workers: a systematic review and meta-analysis Canadian Journal of Infectious Diseases and Medical Microbiology 2021 2021 9019534 10.1155/2021/9019534 33564345 PMC7864758

[12] Tsega D Gintamo B Mekuria ZN Demissie NG Gizaw Z Occupational exposure to HIV and utilization of post-exposure prophylaxis among healthcare workers at St. Peter’s specialized hospital in Addis Ababa, Ethiopia Scientific Reports 2023 13 1 7021 10.1038/s41598-023-34250-4 37120700 PMC10148887

[13] Weldetekle H Teka H Gidey H Sharew A Gebremeskel M Magnitude and determinants of occupational exposure to blood and body fluids among physicians in a teaching hospital in northern Ethiopia Scientific Reports 2025 15 1 10853 10.1038/s41598-025-95301-6 40158014 PMC11954945

[14] Akbulut I Demirci F Exposure of healthcare workers to blood and bodily fluids: a 10-year retrospective analysis at a single center Haydarpaşa Numune Medical Journal 2025 65 1 32 37 10.14744/hnhj.2024.22230

[15] Ippolito G Puro V De Carli G The risk of occupational human immunodeficiency virus infection in health care workers. Italian Multicenter Study. The Italian Study Group on occupational risk of HIV infection Archives of Internal Medicine 1993 153 12 1451 1458 10.1001/archinte.1993.00410120035005 8512436

[16] Allan-Blitz LT Mayer KH Missed opportunities: a narrative review on why nonoccupational postexposure prophylaxis for HIV is underutilized Open Forum Infectious Diseases 2024 11 8 ofae332 10.1093/ofid/ofae332 39086468 PMC11289484

[17] Queiroz AAF Mendes IAC Dias S Barriers to access to HIV post-exposure prophylaxis: a case study Acta Paulista de Enfermagem 2022 35 eAPE039007634 10.37689/acta-ape/2022AO007634

[18] Devred I Kayembe K Valin N Rougier H Shinga BW Prophylaxis by doravirine–lamivudine–tenofovir disoproxil fumarate or elvitegravir–cobicistat–emtricitabine–tenofovir alafenamide after sexual exposure to HIV BMC Infectious Diseases 2023 23 1 578 10.1186/s12879-023-08544-x 37667182 PMC10478445

[19] Mulka L Annandale D Richardson C Fisher M Richardson D Raltegravir-based HIV postexposure prophylaxis (PEP) in a real-life clinical setting: fewer drug-drug interactions (DDIs) with improved adherence and tolerability Sexually Transmitted Infections 2016 92 2 107 10.1136/sextrans-2015-052262 26892929

[20] Gantner P Hessamfar M Souala MF Valin N Simon A Elvitegravir-cobicistat-emtricitabine-tenofovir alafenamide single-tablet regimen for human immunodeficiency virus postexposure prophylaxis Clinical Infectious Diseases 2020 70 5 943 946 10.1093/cid/ciz577 31804669

[21] Gan L Xie X Fu Y Yang X Ma S Safety and adherence of bictegravir/emtricitabine/tenofovir alafenamide for HIV post-exposure prophylaxis among adults in Guiyang, China: a prospective cohort study BMC Infectious Diseases 2024 24 1 565 10.1186/s12879-024-09407-9 38844855 PMC11157740

[22] Gökengin D Korten V Kurtaran B Tabak F Ünal S HIV/AIDS Tanı, İzlem ve Tedavi El Kitabı. Version 3.1 Ankara, Türkiye Nobel Tıp Kitabevleri 2024 978-605-335-979-1 (in Turkish)

[23] Horak J Venter WD Wattrus C Papavarnavas N Howell P Southern African HIV Clinicians Society 2023 guideline for post-exposure prophylaxis: updated recommendations Southern African Journal of HIV Medicine 2023 24 1 1522 10.4102/sajhivmed.v24i1.1522 37795431 PMC10546897

[24] Njemanze U Factors impacting HIV post-exposure prophylaxis among healthcare workers PhD Walden University Minneapolis, MN, USA 2017

[25] Lyatula ZM Ottaru TA Mwanga HH Knowledge on HIV postexposure prophylaxis and associated factors among healthcare workers in Kigoma region, Tanzania Scientific Reports 2025 15 1 31069 10.1038/s41598-025-02513-x 40849316 PMC12375109

[26] Luo Q Luo Y Li T Cui T An integrated online-to-offline model for HIV post-exposure prophylaxis (O2O-PEP) scale-up among men who have sex with men: protocol for developing a pilot randomized controlled trial Frontiers in Public Health 2022 10 1026137 10.3389/fpubh.2022.1026137 36466536 PMC9709450

[27] Johnson LF Lewis DA The effect of genital tract infections on HIV-1 shedding in the genital tract: a systematic review and meta-analysis Sexually Transmitted Diseases 2008 35 11 946 959 10.1097/OLQ.0b013e3181812d15 18685546

[28] Workowski KA Bachmann LH Chan PA Johnston CM Muzny CA Sexually transmitted infections treatment guidelines, 2021 MMWR Recommendations and Reports 2021 70 4 1 187 10.15585/mmwr.rr7004a1 PMC8344968 34292926

[29] Çelik Ekinci S Yenilmez E Yetginakın T Şenyuva A Küçükbirer A Do obstetricians’ knowledge and approaches to HIV and pregnancy reflect the state-of-the-art literature in the era of contemporary ARTs, U=U, and PrEP? Mediterranean Journal of Infection Microbes and Antimicrobials 2025 14 1 9 9 10.4274/mjima.galenos.2025.25386.9

[30] Ceylan M Tol C Emecen AN Kaya A Pullukçu H Hepatitis A screening and vaccination among people living with HIV: when is the ideal time? Mediterranean Journal of Infection Microbes and Antimicrobials 2025 14 1 13 13 10.4274/mjima.galenos.2025.25436.13

[31] Akbulut I Varol ZS Singil S Ödemiş I Atalay S Attitudes, knowledge, and practices of Turkish dentists regarding HIV/AIDS: a cross-sectional study BMC Public Health 2025 25 1 2520 10.1186/s12889-025-23730-z 40691546 PMC12278586

